# NOACs replace VKA as preferred oral anticoagulant among new patients: a drug utilization study in 560 pharmacies in The Netherlands

**DOI:** 10.1186/s12959-017-0156-y

**Published:** 2018-04-18

**Authors:** J. M. van den Heuvel, A. M. Hövels, H. R. Büller, A. K. Mantel-Teeuwisse, A. de Boer, A. H. Maitland-van der Zee

**Affiliations:** 1 Utrecht Institute for Pharmaceutical Sciences (UIPS), Division of Pharmacoepidemiology and Clinical Pharmacology, Utrecht University, Utrecht, The Netherlands; 2 Department of Respiratory Medicine, Academic Medical Center, University of Amsterdam, Amsterdam, The Netherlands; 3 Department of Vascular Medicine, Academic Medical Center, University of Amsterdam, Amsterdam, The Netherlands

## Abstract

**Background:**

In 2012, around 400.000 patients in the Netherlands were treated with Vitamin K Antagonists (VKA) for thromboembolic diseases. Since 2011, non-VKA oral anticoagulants (NOACs) are available. NOACs do not require frequent INR monitoring which benefits patients, but also imposes a risk of reduced therapy adherence. The objective of this study is to describe uptake and patient adherence of NOACs in The Netherlands until October 2016.

**Methods:**

Prescription data for 247.927 patients across 560 pharmacies were used to describe patient profiles, uptake of NOACs among new naive patients and switch between VKA and NOACs, and calculate therapy adherence as the Proportion of Days Covered (PDC).

**Results:**

During the studied period the share of NOACs in oral anticoagulants has grown to 57% of prescriptions to new patients. More than 70% of new NOAC users were new naive patients and around 26% switched from VKA. The overall share of NOACs among starters is largest in the group of patients of 50-80 years. Calculated compliance rate for NOAC patients shows that 88% of all users are adherent with a PDC higher than 80%.

**Conclusions:**

NOAC have overtaken VKA as the major treatment prescribed to new oral anticoagulant patients, and the number of starters on VKA is decreasing. Patients are generally adherent to NOACs during the implementation phase, the period that the medication is used. Fear for inadherence by itself does not need to be a reason for not prescribing NOACs instead of VKA.

## Background

Oral anticoagulants (OAC) are used to prevent and treat a range of thromboembolic diseases. The main indications for oral anticoagulants are atrial fibrillation (AF), venous thromboembolism (VTE) (comprising of deep vein thrombosis (DVT), pulmonary embolism (PE)) and mechanical heart valves [1–3] and for the prevention of thromboembolism after hip or knee replacement surgery [4]. The oral anticoagulants that are currently available in The Netherlands include the vitamin K antagonists (VKA) acenocoumarol and phenprocoumon and the newer oral anticoagulants (dabigatran, rivaroxaban, apixaban, and edoxaban), also called direct oral anticoagulants (DOACs) or non-VKA oral anticoagulants (NOACs) [5]. One NOAC (rivaroxaban) is also registered to be prescribed in triple therapy after acute coronary syndrome (ACS) [5].

In 2012, nearly 400,000 people in the Netherlands were treated with Vitamin K antagonists (VKAs) [4]. VKAs have a small therapeutic window. Treating patients with VKAs requires titration of the dose, and the required dosage can differ largely among patients [6, 7]. If the dose is too low, clots may form in the bloodstream and if the dose is too high, hemorrhages can occur [4]. For this reason International Normalized Ratio (INR) must be frequently monitored to adjust the dose if necessary. For this intensive supervision, a system of Thrombosis Services exists in the Netherlands [4].

Recently, NOACs have proven to be an effective and safe alternative to VKA for prevention of stroke and systemic embolism in patients with AF and patients with VTE [6, 7]. Compared to VKAs, NOACs offer simplification of long-term anticoagulation therapy because they do not require frequent INR monitoring and less frequent dose adjustments. However, also NOACs may require dose adjustments according to age, body weight, renal function and concomitant use of glycoprotein inhibitors [8]. Absence of frequent monitoring may lead to an increased risk of undetected reduced therapy adherence, with potentially severe consequences [6].

Up until now, it is not known what the uptake of NOACs in the Netherlands is. The aim of the present study is therefore to describe uptake and patient adherence of the NOACs dabigatran, rivaroxaban, apixaban and edoxaban in The Netherlands between July 2011 and October 2016, based on pharmacy prescription data.

The following research questions are addressed: how many patients are treated with oral anticoagulants, and what is the percentage that receives NOACs? How many patients are newly initiated on NOACs? What is the impact of the introduction of NOACs on the usage of VKA? Are there patients already treated with VKA that switch to using NOACs? Are there differences in characteristics between patients that use VKA and patients that use NOACs? Finally, are patients therapy adherent during the period in which they are treated with a NOAC?

## Methods

### Data collection and study population

For this study, data from the NControl database were obtained. Our dataset contains data of 544 pharmacies, spread across The Netherlands with data for the complete study period. The total number of public pharmacies in The Netherlands is approximately 1900. Since 2011, the NControl database contains data related to over 557 million prescriptions and 7.2 million patients.

The database contains (not exhaustive) the following information about the prescriptions, the dispensed medication and quantity, dispensing date, prescribed daily dosage, prescriber type and the patient’s age and gender. Patients in the database cannot be identified, but can be tracked over time across pharmacies in the database. Prescribers are anonymized and cannot be identified nor tracked over time. NControl is allowed to use these prescription data for research purposes. NControl adheres to data protection and privacy regulations, as established in amongst others the Personal Data Protection Act in The Netherlands as well as the Netherlands Norm (NEN) 7510 standard, related to information protection in healthcare, which is derived from International Organisation for Standardization (ISO) norm 27,001 and 27,002. Because most patients in The Netherlands are registered with a single community pharmacy, dispension records in the Ncontrol database contain a (virtually) complete view of patient history of prescription drugs [9].

All patients who received at least one prescription for VKAs or NOACs between 1 July 2011 and 30 September 2016 were included in the study.

### Data analysis

We analysed the uptake of NOACs versus VKAs by measuring the number of prescriptions per month. The proportions of NOACs and VKAs in the total amount of prescribed anticoagulants and the proportion prescribed to new naive patients were calculated separately. A naive starter was defined as a patient who received a first prescription for an OAC, and had a history of other medications in the NControl database for at least 1 year before this OAC initiation. Any other first OAC prescription has been excluded from the analyses of new naive patients.

A patient who did not receive an OAC for 365 days or more, is labelled a stopper. We took 365 days to limit misclassification of inadherent patients or patients that missed a prescription at the public pharmacy e.g. because of hospitalization as a stopper.

For any patient a switch was defined as a first prescription for an OAC in a particular anatomical, therapeutical and chemical (ATC) medication cluster that was preceded by an anticoagulant drug from another ATC cluster. There are four switching categories: switch from VKA to NOAC, from NOAC to VKA, between VKAs and between NOACs.

We analyzed if prescribers of NOACs targeted specific patient groups in terms of age, gender and number of co-medications used. This number was used as a proxy for a patient’s general health status.

Therapy adherence consists of three phases: initiation, implementation and discontinuation [10]. We specifically looked that therapy adherence during treatment with NOACs, i.e. the implementation phase. For this reason we calculated adherence as the Proportion of Days Covered (PDC) [11, 12]. In literature, more than one definition for PDC can be found [11–15]. Our definition of PDC can also be referred to as Compliance Ratio (CR) [13, 14]. This metric is calculated through the following formula:

$$ PDC=\frac{100\%\ast \mathrm{Number}\ \mathrm{of}\ \mathrm{days}\ \mathrm{of}\ \mathrm{supply}\ \left(\mathrm{excl}.\kern0.5em \mathrm{last}\ \mathrm{dispension}\right)}{\mathrm{Number}\ \mathrm{of}\ \mathrm{days}\ \mathrm{between}\ \mathrm{first}\ \mathrm{and}\ \mathrm{last}\ \mathrm{dispension}} $$

The number of days of supply equals the quantity dispensed divided by the daily dosage indicated on the prescription. To calculate PDC, we only included patients with dispensions on two or more different dates. Patients that did not get a refill after their first NOAC dispension were excluded from the adherence analyses. We also excluded patients that received weekly dispensions, because PDC for these patients will always be close to 100%. Patients with a PDC of 80% or higher are considered adherent. This cut-off is supported by the International Society for Pharmaceutical and Outcomes Research [15].

Differences between groups were tested by analysis of variance (ANOVA) and t-tests.

For analysis and reporting SQL server 2014 and Excel 2013 were used.

Unless mentioned otherwise, year totals refer to the period of the 12 months ending 1 October of that year, e.g. 2016 means 1 October 2015 to 30 September 2016.

## Results

The total number of OAC users included in this study was 256,641 across 544 pharmacies and they received a total of 3,029,294 VKA or NOAC prescriptions between 1 July 2011 and 30 September 2016.

### Uptake

In our panel, the total number of patients on OACs grew from 126,638 in 2012 to 159,291 in 2016, with an average yearly growth rate of 5.9%. The number of naive starters per month oscillated around an average of 2553.

**Fig. 1:**
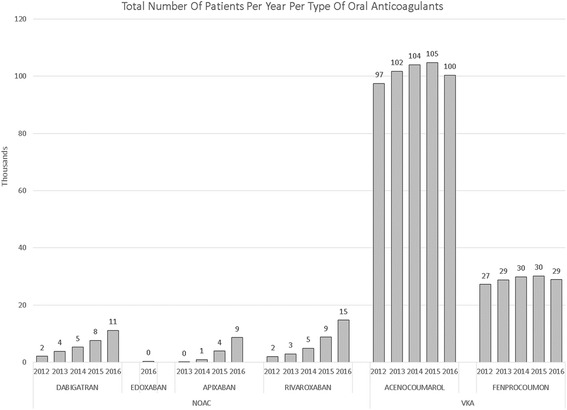
Total number of patients per year per type of oral anticoagulant

**Fig. 2:**
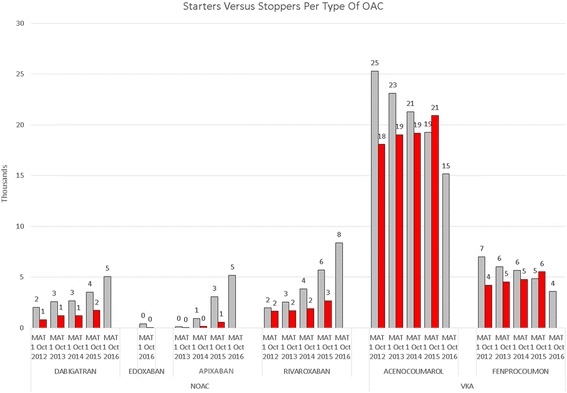
Starters versus stoppers per type of oral anticoagulant

**Fig. 3:**
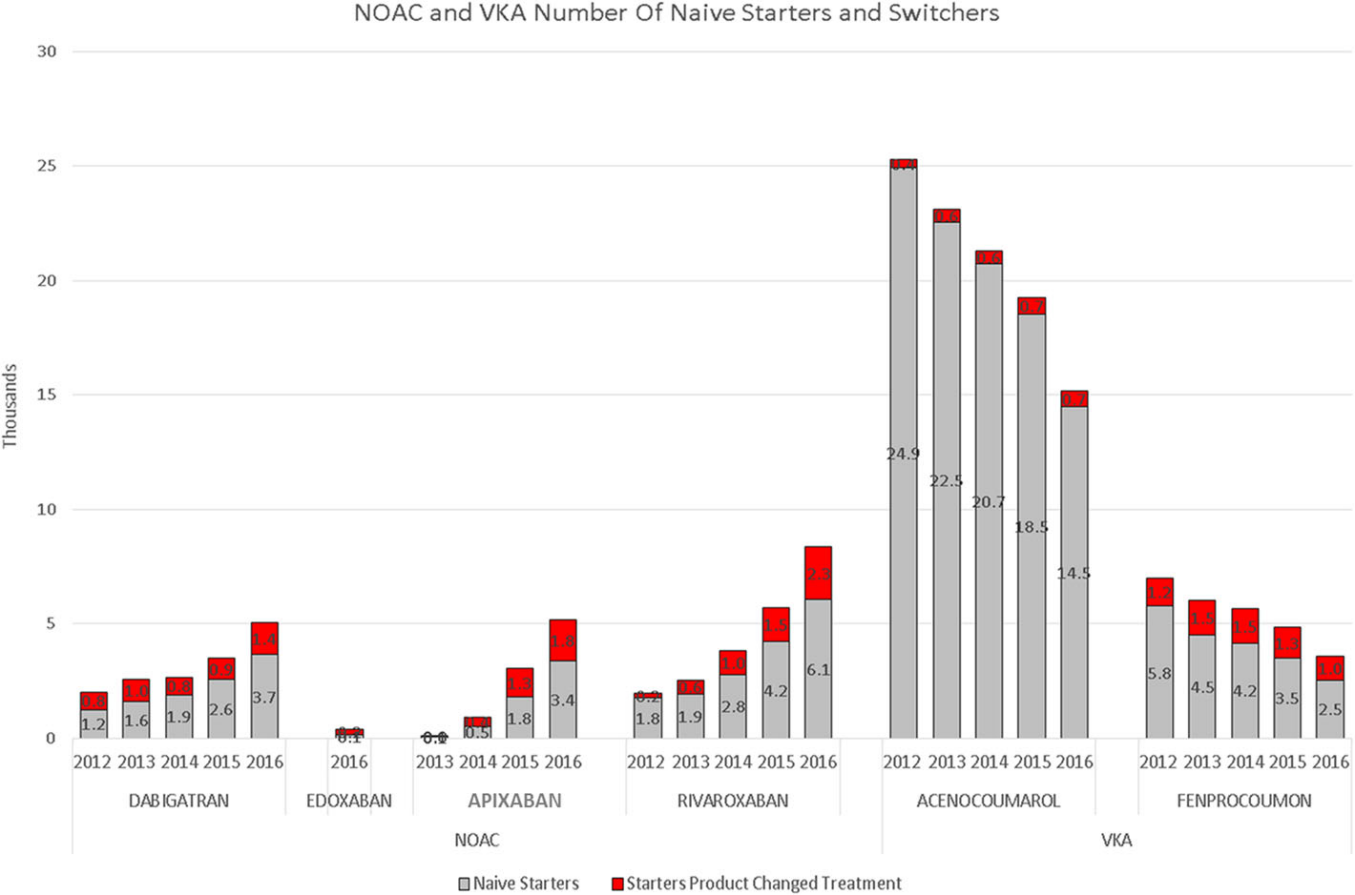
Number of naive starters and switchers for NOAC and VKA

**Fig. 4:**
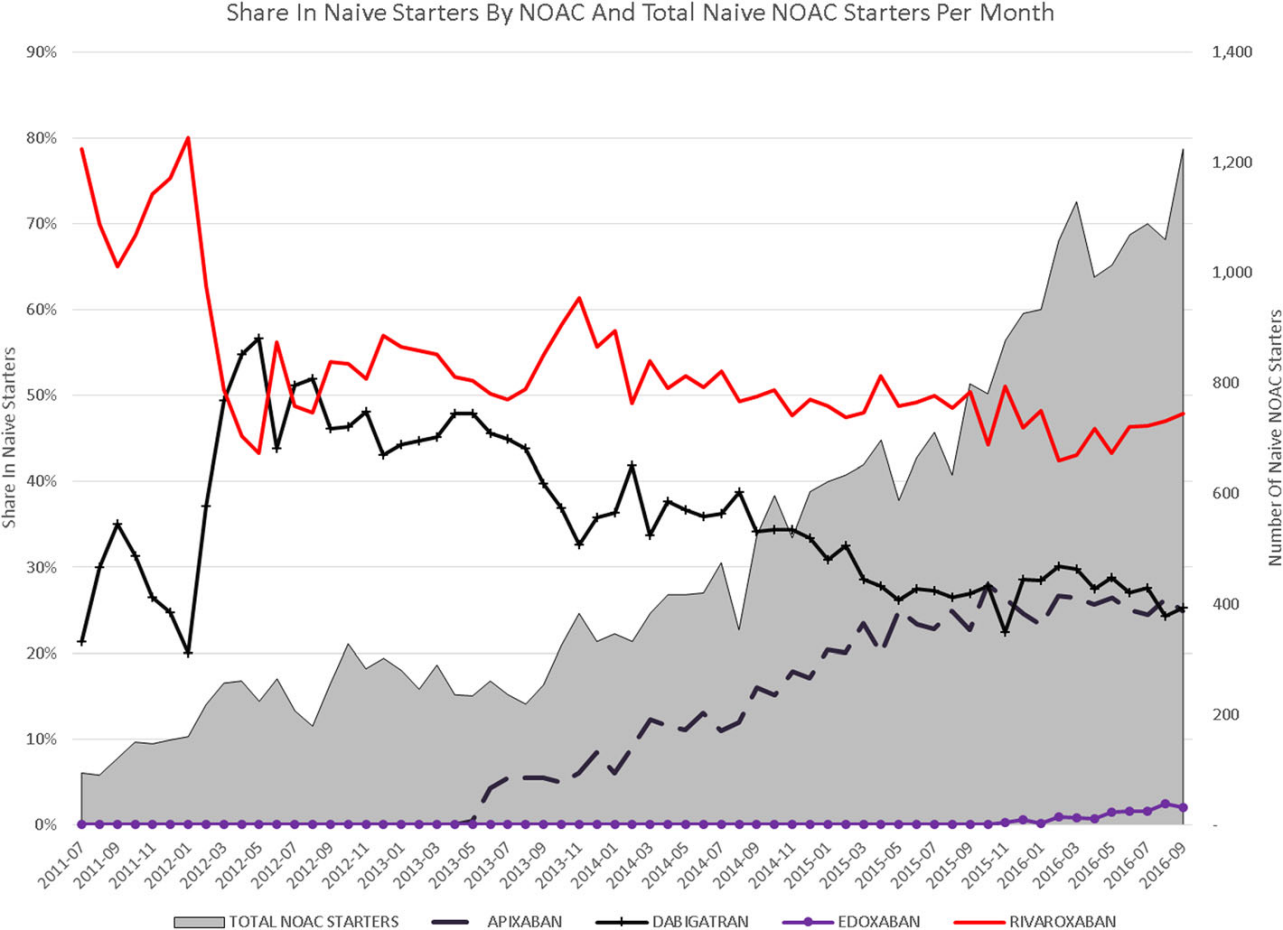
Share in naive starters per NOAC and total naive NOAC starters per month

**Fig. 5:**
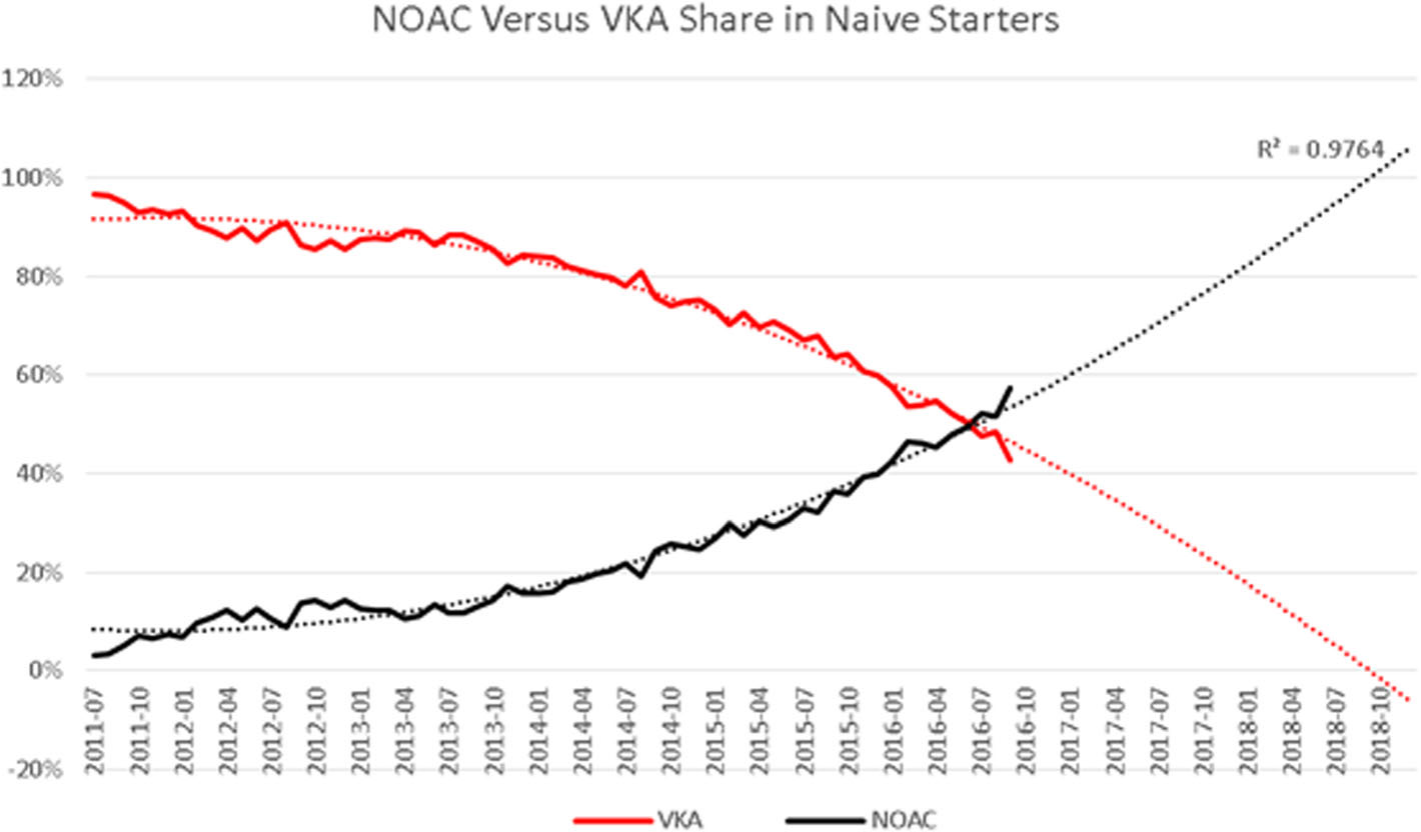
Trend in NOAC and VKA Share in naive starters

During the past 5 years, the number of NOAC starters has increased steadily, from 4000 in 2012 to 40,000 in 2016. Rivaroxaban is most used in terms of absolute patients, followed by dabigatran and apixaban. However, apixaban has the fastest growth rate (119% in 2016) and an equal number of naive starters as dabigatran in 2016 (Figs. 1, 2 and 3). In recent months, the share of dabigatran and apixaban in naive starters on NOAC each oscillate around 25%. From June 2013, when apixaban received a reimbursement status for the AF indication, the total number of naive starters on NOACs also started to grow and apixaban quickly gained market share from rivaroxaban and mainly dabigatran. The share of dabigatran in naive starters stabilized from June 2015, when the indications for dabigatran were extended from AF and the prevention of VTE after knee or hip replacement surgery to include also DVT and PE. Since the introduction of apixaban, the share of rivaroxaban in naive starters has also declined somewhat, but less than that of dabigatran, and remained relatively stable until January 2016, when the share of dabigatran in naive starters slightly increased again (Fig. 4). Overall, the share of NOACs in naive starters is growing at an increasing speed and was 57% in September 2016 (Fig. 5).

For VKA, during this same studied period, we have seen the number of patients grow at a declining pace up until 2015. In 2016 the number of VKA patients declined for the first time. These last 12 months, the population of VKA patients decreases with 6000 patients (−4.4%) (Fig. 1). In 2015, the number of patients stopping VKA treatment is increasing, while the number of starters in 2016 decreases, resulting in a net decline of patients (Fig. 3). (We have no information on stoppers for 2016 yet: we need 365 days after a last dispensing to label a patient as stopper.)

### Source of NOAC patients

During the studied period, 48,291 patients started with a NOAC. 70% of this group where new naive patients and 30% were switchers from either VKA or another NOAC. A total of 12,769 (26% of NOAC starters) were patients switching from VKA. In comparison, during that same period, 3437 patients switched from a NOAC to VKA. 2480 patients switched between NOACs.

### Patient targeting

To analyze if prescribers of NOACs targeted specific patient groups, age, gender and the number of co-medications used were extracted. OACs were mainly prescribed to adults. During the study period, there were only 181 initiations of OACs to patients under the age of 18, and only 6 of those (3.3%) received a NOAC.

**Fig. 6:**
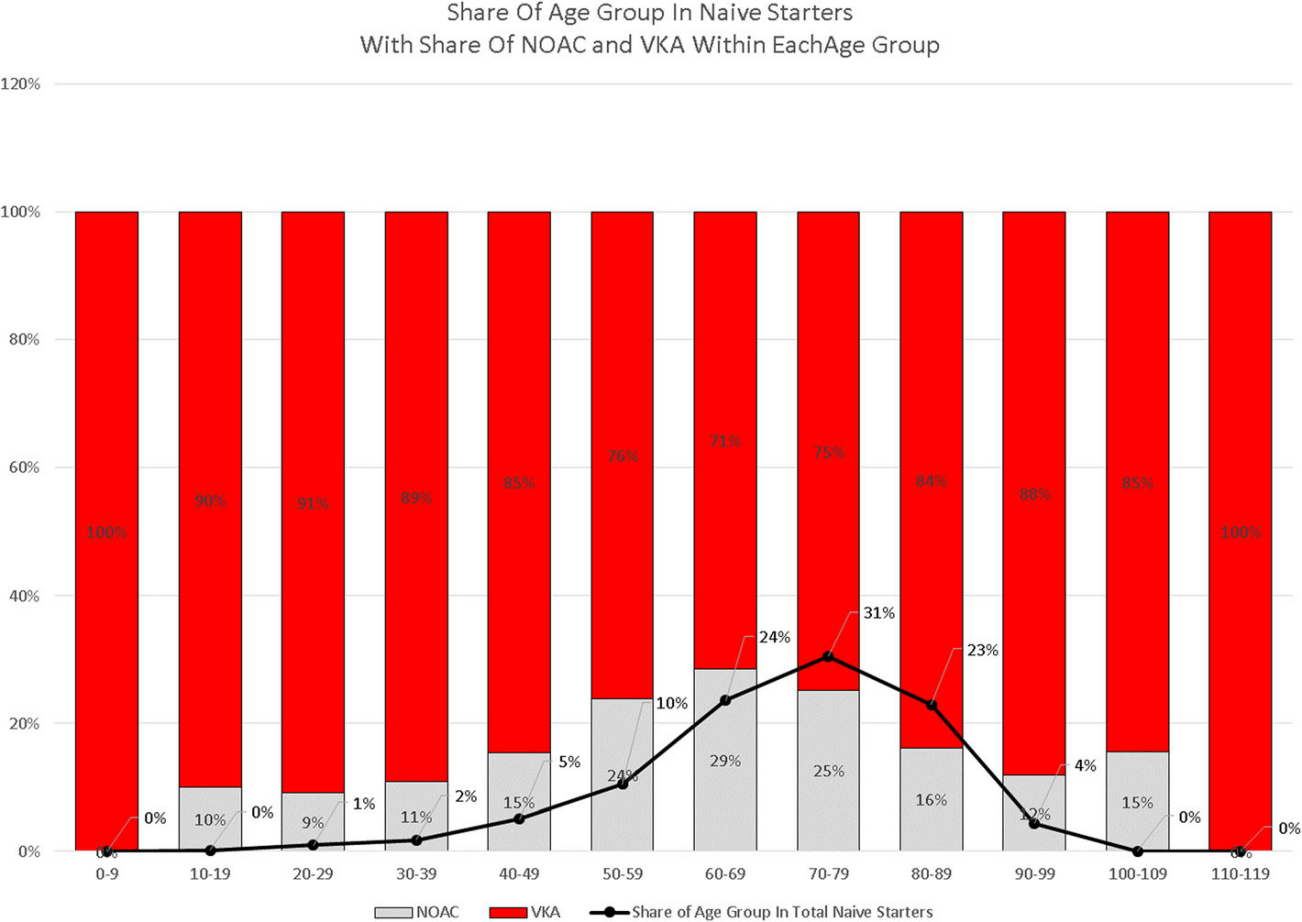
Share of age groups in naive starters and share of NOACs in age groups

The proportion of NOAC prescriptions was highest (29%) in the group of new users aged between 60 and 69, whereas the average proportion of NOACs in naive starters on OACs was 22% (Fig. 6). Share of NOACs in naive starters was also above average in the age groups 50-59 (24%) and 70-79 (25%).

The share of female starters in NOACs was 48% on average. However this proportion differed somewhat across age categories: the largest group of females (34% of all females) starting on NOACs was between 70 and 79 years old. This was also the age group with most starters on OACs in general. In the group of patients aged 80 and older, share of women was over 60%, and in the group below 70, they represented 44%. The largest group of male starters was aged between 60 and 69 (34%) (Table 1) (Fig. 6).

**Table 1:** Patients by age and gender

Age group	NOAC					VKA				
	Male		Female		Total	Male		Female		Total
	Nr of patients	pct in age group	Nr of patients	pct in age group	Nr of patients	Nr of patients	pct in age group	Nr of patients	pct in age group	Total
0-9						34	59%	24	41%	58
10-19	14	41%	20	59%	34	117	36%	206	64%	323
20-29	66	46%	76	54%	142	386	29%	925	71%	1311
30-39	170	53%	148	47%	318	950	40%	1451	60%	2401
40-49	762	59%	539	41%	1301	3276	49%	3343	51%	6619
50-59	2343	58%	1671	42%	4014	7691	64%	4297	36%	11,988
60-69	5656	54%	4791	46%	10,447	15,638	62%	9759	38%	25,397
70-79	5228	49%	5428	51%	10,656	17,509	54%	15,211	46%	32,720
80-89	1906	40%	2831	60%	4737	10,212	40%	15,063	60%	25,275
90-99	193	33%	389	67%	582	1100	27%	3039	73%	4139
100-109	1	17%	5	83%	6	7	19%	29	81%	36
110-119						1	50%	1	50%	2
Total	16,339	51%	15,898	49%	32,237	56,921	52%	53,348	48%	110,269

On average, VKA patients used 9.4, and NOAC users used 8.2 different medications during the 6 months before their first oral anticoagulant prescription (*p* < 0.0001)). There was a difference across age categories: 8.6 different medications for NOAC patients versus 10.0 for VKA patients aged above 65 (p < 0.0001), and 7.0 ATCs for NOAC starters versus 7.6 for VKA starters aged below 65 (p < 0.0001).

### Adherence

88% of NOAC users had a PDC above 80% (Table 2). Mean PDC was 108% for dabigatran, 107% for apixaban and 112% for rivaroxaban (Table 3). Only the PDC for rivaroxaban was significantly different to the mean of dabigatran (*p* = 0.0026) and to the mean of apixaban (*p* = 0.0331). The mean PDC for apixaban and dabigatran were not significantly different.

**Table 2:** Percentage of adherent patients per NOAC

	Pct of patients	
	PDC* > =80	Nr of patients
Apixaban	92%	7094
Dabigatran	88%	11,782
Rivaroxaban	88%	13,975
Total	89%	32,851

**Table 3:** Average PDC per NOAC

PDC^a^	Apixaban	Dabigatran	Rivaroxaban
Mean	109	108	113
Std.dev.	81	141	120
Median	102	101	103

^a^ All patients with more than 1 dispensing

Excluding patients with weekly dispensings

## Discussion

In The Netherlands, NOACs are increasingly prescribed and gained market share from VKA among existing and especially new patients. Patients treated with NOACs are in general adherent to their therapy, during the implementation phase of their treatment. NOACs represented 57% of all new prescriptions in the month of September 2016, even though the introduction of NOACs in the Netherlands has met some resistance, [4, 16, 17]). The introduction has been gradual, as was advised by the Health Council of the Netherlands, but now the share of NOACs in starters increases every month, and the number of VKA patients is decreasing since 2016. If the current trend continues, within 24 months we expect virtually all naive starters to receive a NOAC, and that only a limited number of patients with a contra-indication for NOAC will still start on VKA. We did not find evidence that NOACs were targeted to specific patient groups, except for some specific age groups. The fact that patients treated with NOACs are generally adherent to their therapy during the implementation phase is specifically important because unlike VKA patients, they are not under continuous supervision of the Thrombosis Service in The Netherlands. The therapy adherence that we measured is high compared to adherence to other medications measured in other studies [12] and in line with high adherence to NOACs in other comparable studies [14, 18, 19]. Borne et al. [18] use a comparable method for calculating PDC, only exclude patients with less than 1 year follow-up and find that 74.2 patients have a PDC > = 80%. Schulman et al. use the same method for calculating PDC and arrive at 89% of all patients with a PDC > = 80% [19]. Mueller et al. find that 90.6% of all patients have a compliance ratio of >80% [14], also using this method of calculation. Our method, as is the case for the above mentioned studies, only includes patients that received more than one dispension. Also, the period for which the last dispension was valid was disregarded. As a result, (early) discontinuation (cessation) does dot not impact the score. This may lead to an upward bias in adherence scores, compared to calculation methods that take a fixed time interval after starting a medication to calculate PDC, like the study by Maura et al. [20] and many of the studies that are summarized in the review by Obamiro et al. [21] and report lower adherence.

We chose our method because we have no knowledge of the indication, and do not know with certainty how long a patient is supposed to use the prescribed NOAC, and because we wanted to assess compliance explicitly during treatment, excluding the impact of discontinuation.

Like other studies of medication adherence, our study is also limited by the accuracy of assessing adherence from pharmacy prescription data, which may misclassify the adherence of patients who fill prescriptions but do not actually take them.

Multiple studies have shown that the use of NOACs appears to be as efficacious as and safer than the use of VKAs [6, 7] and the number of NOAC prescriptions is increasing also in other countries. From other national level database studies, we know that the number of NOAC prescriptions in Canada increased annually between 2008 and 2014, from 1% to 33% of all OAC prescriptions [22]. In the US, NOACs have had a modest but growing uptake (from 0.04 in the beginning of 2011 to 12% in second quarter of 2012) among atrial fibrillation patients hospitalized with stroke or transient ischemic attack [23]. Other, smaller studies have shown an increasing uptake of NOACs among patients using oral anticoagulants in specific hospitals [24, 25].

Some arguments mentioned against introducing NOACs in The Netherlands state that with the Thrombosis Services, The Netherlands has an excellent infrastructure to monitor patients using VKA, and the use of NOACs in clinical practice has both positive and negative aspects. Initially, in the media and in politics, there has been criticism around the benefits of NOACs over VKA. Until recently no antidote for NOACs was available - idarucizumab, an antidote for dabigatran was introduced only in January 2016 - and the benefit that NOACs are to be used without constant supervision of the Thrombosis Service could be countered by the argument that this reduced supervision could lead to a lower therapy adherence, accompanied with health risks. Also, in the period following the introduction of dabigatran and rivaroxaban, NOACs met opposition for reasons associated with healthcare budgets [16] and increased risk of gastrointestinal bleeding [17], contrary to the results of more recent studies [6, 7]. This may have hampered the uptake of NOACs. Our study showed that NOAC users are generally very therapy adherent, which could be an argument in favor of prescribing NOACs in the future. We found that the largest percentage of adherent patients was found under rivaroxaban users. Even though our t-tests showed statistically significant differences with dabigatran and apixaban, we believe that these are not clinically relevant.

Dabigatran was the first NOAC on the market and had the largest market share during the first period after its introduction, but it lost market share first to rivaroxaban, and then to apixaban. We observed an immediate start in uptake of apixaban from June 2013, when it received reimbursement status for AF, and since then apixaban gained share almost every month until June 2015. We suspect that the steady growth of apixaban, introduced years after dabigatran and rivaroxaban, was caused at least partly because it was welcomed as a perceived safe alternative to the existing NOACs on the market. Apixaban, like dabigratran needs to be taken twice per day, but has no contra-indication for patients with kidney deficiency, and may therefore also be considered a safer alternative to dabigatran [5]. In this context of the perceived safety of specific NOACs, it is also interesting to note that the share of dabigatran in naive starters increased in January 2016, after the introduction of idarucizumab, its antidote, albeit only for a few months.

Approximately three quarters of patients starting on NOACs were new naive patients. 27% of NOAC starters have switched from VKA and the number of switchers from VKA to NOAC was 3.5 times higher than the number of patients switching from NOAC to VKA, resulting in a net flow of patients from VKA to NOAC. The number of patients switching between NOACs was smaller than the number of patients switching from NOAC to VKA. This suggests that patients experiencing problems caused by their treatment with a NOAC were more likely to switch (back) to VKA than to try another NOAC. The reason for switching back to VKA cannot be explained by our data, but may partly be the result of unfamiliarity of general practitioners with NOACs. Until October 2016, NOACs could only be prescribed by medical specialists. Starting November 2016, also GPs can prescribe NOACs. This might result in an additional acceleration of the speed of uptake of NOACs.

It does not appear that physicians were targeting a specific group of patients in terms of age and gender. Based on the number of co-medications used, it cannot be concluded that NOAC starters were in a significantly better or worse state of health than starters on VKA. The reason why the percentage of patients starting NOACs above the age of 74 was lower may be associated with co-morbidities (among others renal insufficiency and higher bleeding risk, higher risk of falling) [26], even though a meta-analysis clearly showed that those above 75 years of age mostly benefit from using NOACs, both in terms of efficacy and safety [6].

We acknowledge that there are some limitations that may have influenced the results. Pharmacies included in this study are only public pharmacies, no hospital pharmacies. Outpatient pharmacies (4% of all public pharmacies in The Netherlands) were underrepresented. Patients that start NOACs in the hospital may appear only in our panel after discharge. Adherence results can be negatively affected by patients that spend time in a hospital between receiving dispensions from their public pharmacy. Also related to adherence, we analysed adherence only during the period that NOAC was used: the implementation phase. The impact of early cessation is not included in our metric which may lead to a higher calculated adherence. Another limitation is our inability to analyse the reasons for prescribing medication and therefore we have not been able to describe the uptake of NOACs for different indications. We used the number of ATCs prescribed to a patient to infer the general health status of that patient, and we acknowledge that this number by itself is no firm measure of health status, however, it is the only information available to us. Lastly, we analyzed the medication that was dispensed to the patient only. We do not know with certainty whether the patient took all received medication. An important strength of our study is that the population, with almost one third of all public pharmacies in The Netherlands is very large. We consider it representative for public pharmacies in The Netherlands as a whole.

NOACs have gained a solid position in the market in The Netherlands. At present, the majority of new patients are prescribed NOACs, and some VKA users switch to NOACs. NOACs are being used across all adult patient groups in terms of age, gender and health status. We expect that almost all oral anticoagulants prescribed to new patients will be NOAC, even though a number of (new) patients on VKA will likely remain.

The high therapy adherence measured among patients that use NOACs should be considered one of the most relevant outcomes of this study. At the introduction of NOACs in The Netherlands, fear of patients not being adherent to their treatment and the related health risks as a result of absent supervision of NOAC patients by the thrombosis service has been the most important caveat of the Health Council of the Netherlands, related to the prescription of NOACs. Based on our results, fear for inadherence by itself does not need to be a reason for not prescribing NOACs instead of VKA. However, monitoring adherence and identifying (early) discontinuation should remain important.

## Conclusions

NOAC have overtaken VKA as the major treatment prescribed to new oral anticoagulant patients, and the number of starters on VKA is decreasing. Patients are generally adherent to NOACs during the implementation phase, the period that the medication is used. Fear for inadherence by itself does not need to be a reason for not prescribing NOACs instead of VKA.

## Abbreviations

ACS: Acute coronary syndrome
AF: Atrial fibrillation
ANOVA: Analysis of variance
ATC: Anatomical, therapeutical and chemical medication cluster
CR: Compliance ratio
DOAC: Direct oral anticoagulants
DVT: Deep vein thrombosis
INR: International normalized ratio
ISO: International organisation for standardization
NEN: Netherlands Norm
NOAC: Non-VKA oral anticoagulants
OAC: Oral anticoagulants
PDC: Proportion of days covered
PE: Pulmonary embolism
VKA: Vitamin K Antagonists
VTE: Venous thromboembolism
